# Neuropathic Pain Causes Pyramidal Neuronal Hyperactivity in the Anterior Cingulate Cortex

**DOI:** 10.3389/fncel.2018.00107

**Published:** 2018-04-20

**Authors:** Ruohe Zhao, Hang Zhou, Lianyan Huang, Zhongcong Xie, Jing Wang, Wen-Biao Gan, Guang Yang

**Affiliations:** ^1^Langone Medical Center, Neuroscience Institute, New York University School of Medicine, New York University, New York, NY, United States; ^2^Langone Medical Center, Department of Neuroscience and Physiology, Skirball Institute, New York University School of Medicine, New York University, New York, NY, United States; ^3^Department of Anesthesiology, Perioperative Care and Pain Medicine, New York University School of Medicine, New York University, New York, NY, United States; ^4^Geriatric Anesthesia Research Unit, Department of Anesthesia, Critical Care and Pain Medicine, Massachusetts General Hospital and Harvard Medical School, Charlestown, MA, United States

**Keywords:** anterior cingulate cortex (ACC), acute pain, neuropathic pain, two-photon imaging, pyramidal neurons

## Abstract

The anterior cingulate cortex (ACC) is thought to be important for acute pain perception as well as the development of chronic pain after peripheral nerve injury. Nevertheless, how ACC neurons respond to sensory stimulation under chronic pain states is not well understood. Here, we used an *in vivo* two-photon imaging technique to monitor the activity of individual neurons in the ACC of awake, head restrained mice. Calcium imaging in the dorsal ACC revealed robust somatic activity in layer 5 (L5) pyramidal neurons in response to peripheral noxious stimuli, and the degree of evoked activity was correlated with the intensity of noxious stimulation. Furthermore, the activation of ACC neurons occurred bilaterally upon noxious stimulation to either contralateral or ipsilateral hind paws. Notably, with nerve injury-induced neuropathic pain in one limb, L5 pyramidal neurons in both sides of the ACC showed enhanced activity in the absence or presence of pain stimuli. These results reveal hyperactivity of L5 pyramidal neurons in the bilateral ACC during the development of neuropathic pain.

## Introduction

Acute pain is a physiological response vital for the organism to avoid potential tissue damage. The neural pathway for acute pain involves peripheral nociceptors, spinal cord, brain stem, thalamus and a variety of cerebral structures, including somatosensory, insular and anterior cingulate cortices (Talbot et al., 1991; Casey, 1999). Pain perception is thought to occur in the anterior cingulate cortex (ACC; Rainville et al., 1997; Vogt, 2005) which receives nociceptive inputs from the medial thalamus (Robertson and Kaitz, 1981; Vogt and Sikes, 2000) and reciprocally connects with a variety of cortical regions (Shyu et al., 2010). Studies in humans have consistently reported that painful body stimuli evoke electrical potentials (Lenz et al., 1998) and metabolic activation (Lenz et al., 1998; Ploner et al., 2002; Wager et al., 2013) in the ACC, the degrees of which are correlated with the intensity of pain (Coghill et al., 1999; Büchel et al., 2002). At the cellular level, nociceptive responses from ACC neurons have been demonstrated by *in vivo* electrophysiological recordings in anesthetized mice (Koga et al., 2010), rats (Yamamura et al., 1996) and rabbits (Sikes and Vogt, 1992; Shyu et al., 2010), as well as in conscious, behaving animals (Koyama et al., 1998, 2001; Wang et al., 2003; Kuo and Yen, 2005; Zhang et al., 2011, 2017; Chen et al., 2017). Furthermore, lesions or inactivation of the ACC have been shown to relieve aversive responses to acute pain (Foltz and White, 1962; Lubar, 1964; Ballantine et al., 1967; Turnbull, 1972; Zhang et al., 2017).

Despite the prominent function of the ACC in acute nociception, the contribution of the ACC to the experience of chronic pain remains unclear. Neuropathic pain is a chronic pain state that arises from peripheral or central nerve injury (Baron, 2006). Recent evidence indicates that with neuropathic pain, cortical neurons in layer 2/3 (L2/3) and layer 5 (L5) of the ACC undergo various alterations, including enhanced excitatory synaptic neurotransmission (Zhao et al., 2006; Xu et al., 2008; Li et al., 2010; Koga et al., 2015; Bliss et al., 2016), dendritic dysfunction (Santello and Nevian, 2015), increases in intrinsic cellular excitability and decreases in inhibition (Blom et al., 2014). All of these changes profoundly influence neuronal firing, contributing to central sensitization and pathological pain responses (Wu et al., 2005; Koga et al., 2015). Because neurons in the ACC are activated in both acute and chronic pain states (Bliss et al., 2016), it is imperative to examine and identify neuronal responses that could discriminate between acute and chronic pain. Such knowledge is important for the development of interventions to relieve chronic pain while minimally affecting acute nociception.

The purpose of the current study is to use *in vivo* two-photon microscopy to investigate the activity of L5 pyramidal neurons, the principal output neurons of the ACC, under both acute and chronic pain conditions. By recording calcium activities of pyramidal neurons in awake mice, sensory stimulation-evoked neuronal activity was revealed without the confounding effects of general anesthesia. We found that a significant number of L5 pyramidal neurons in the ACC responded to acute noxious stimuli with an immediate rise of somatic Ca^2+^ transients. These neuronal responses correlated with noxious intensities and occurred bilaterally to noxious stimuli applied to either contralateral or ipsilateral hind paws. In mice with neuropathic pain, L5 neurons showed an increase in both spontaneous and noxious stimuli-evoked activities in the bilateral ACC. Together, our studies provide the first *in vivo* characterization of pain-related calcium activities of L5 pyramidal neurons in the ACC, and suggest that the development of neuropathic pain is accompanied by the hyperexcitability of the bilateral ACC.

## Materials and Methods

### Animals

All experiments were performed in accordance with the National Institutes of Health guidelines and regulations. The animal protocol was approved by the New York University School of Medicine Animal Care and Use Committee. *Thy1*-GCaMP6s mice (Founder Line 3) expressing GCaMP6s in cortical pyramidal neurons were generated in the laboratory of Dr. Wen-Biao Gan. Mice were group-housed in the New York University Skirball animal facility. Two to three-month-old mice of both sexes were used in this study in order to include sex as a potential biological variable. Because the sex of the mice had no effect on pain-related neuronal responses in the ACC, data from male and female mice were grouped together. An average of four mice per group was used in Ca^2+^ imaging experiments.

### Surgical Preparation for Imaging Awake, Head-Restrained Mice

*In vivo* Ca^2+^ imaging was performed in awake, head-restrained mice. The surgical procedure for preparing awake animal imaging has been described previously (Yang et al., 2013; Cichon et al., 2017). In short, while the animal was under deep anesthesia via an intraperitoneal injection of ketamine (100 mg/kg) and xylazine (15 mg/kg), a midline incision of the scalp was made to expose the periosteum, and the skull above the bilateral ACC was located based on stereotactic coordinates (+0.5–1.0 mm anterior of bregma and 0.3–0.8 mm lateral to midline; Allen Mouse Brain Atlas) and marked with ink. A head holder composed of two parallel micro-metal bars was attached to the animal’s skull with glue (Loctite 495) to help restrain the head and reduce motion-induced artifacts during imaging. The head holder was further fortified with dental acrylic cement. After the cement was completely dry, the marked skull region was thinned with a dental drill to a thickness less than 30 μm and then peeled off with the surgical forceps. Dura over the exposed cortex was partially removed. Precaution was taken to avoid tearing the dura on top of the superior sagittal sinus and bridging veins. A glass coverslip (approximately the same size as the bone being removed) was glued to the skull, over the exposed cortex. The surrounding area of the glass window was fortified with dental cement. Mice were given at least 24 h to recover from the surgery-related anesthesia. Before imaging, mice with head mounts were habituated to the imaging apparatus three times (10 min each) to minimize potential stress effects of head restraining and imaging. No obvious distress was observed in habituated animals during imaging experiments.

### *In Vivo* Ca^2+^ Imaging

To image neuronal Ca^2+^ activity in the cortex of awake mice, the head holder was screwed to two metal cubes attached to a solid metal plate. Together with the metal plate, the head-restrained animal was placed on the stage of a two-photon microscope. The *in vivo* Ca^2+^ imaging experiments were performed using a Bruker two-photon laser scanning system equipped with a Ti:sapphire laser (Mai Tai DeepSee; Spectra Physics) tuned to 890 nm. To identify L5 of the ACC, an image stack of neurons within a depth of 900 μm from the pial surface was collected to generate a 3-dimensional map and compared with the sagittal atlas. Ca^2+^ images were collected from the dorsal part of the ACC (AP: +0.5–1.0 mm, ML: 0.3–0.8 mm, DV: 0.75–0.9 mm) while the animal was under a quiet resting condition, as well as when a mechanical stimulation was applied to the contralateral or ipsilateral hind paw. The average laser power on the brain sample was ~20–30 mW. All experiments were performed using a 25× objective immersed in an artificial cerebral spinal fluid (ACSF) solution and with a 1× digital zoom. Images were collected at a resolution of 512 × 512 pixels and a frame rate of 2 Hz. Image acquisition was performed using Bruker PrairieView v.5.4. software. After Ca^2+^ imaging, a red dye was intracerebrally injected to the imaged area and the animal was fixed with 4% paraformaldehyde. The brain was then sectioned at 200 μm thickness and imaged using confocal microscopy to confirm that the dye-labeled region was within the ACC.

### Analysis of Imaging Data

Imaging data were analyzed *post hoc* using NIH ImageJ software. When the animal was in a quiet resting state, motion-related artifacts due to the animal’s respiration and heart beat were typically less than 2 μm as detected in our cortical measurements. Vertical movements were infrequent and minimized by habituation and the use of head holder attached to the animal’s skull by dental acrylic. If the animal struggled on the stage of the microscope, imaging time points from those segments were excluded from quantification. Δ*F*/*F*_0_ was calculated as (*F* – *F*_0_)/*F*_0_, where *F*_0_ is the baseline fluorescence signal averaged over a 2-s period corresponding to the lowest fluorescence signal over the recording period. GCaMP6 fluorescence has a long decay time constant (Chen et al., 2013), therefore it is difficult to report firing rates based on calcium responses. To compare neuronal activity under various behavioral states (with or without peripheral mechanical stimuli) and among different cells, we performed an integrated measurement of a cell’s output activity over 40-s recording, termed total integrated calcium activity. The total integrated calcium activity was the average of Δ*F*/*F*_0_ over 10 s per trial (a total of four trials). Neurons responsive to peripheral stimulation were defined as the cells in which evoked somatic activity was beyond three standard deviations of the mean baseline activity.

### Spared Nerve Injury

Spared nerve injury (SNI) of the sciatic nerve or sham operation was performed under strict sterile conditions (Decosterd and Woolf, 2000; Bourquin et al., 2006; Cichon et al., 2018). In short, adult mice (2–3 months old) were deeply anesthetized with an intraperitoneal injection of ketamine (100 mg/kg) and xylazine (15 mg/kg). A small incision was made in the left or right thigh to expose the sciatic nerve and its peripheral branches (common peroneal, tibial and sural nerves). The 8/0 nylon thread (S&T, Fine Science Tools, CA, USA) was slipped under the common peroneal and the tibial nerves to make a ligation and cut, leaving the sural nerve intact. Great care was taken to avoid any contact with or stretching of the intact sural nerve. Muscle and skin were closed in two layers. For sham surgery, the sciatic nerve was exposed but not ligated or cut.

### Withdrawal Threshold Test

Mechanical nociceptive threshold was assessed using von Frey (vF) filaments. Mice were individually placed into a plexiglass chamber over a mesh table and habituated for 20 min before testing. Beginning with 0.008 g, vF filaments in a set with logarithmically incremental stiffness (0.02, 0.04, 0.07, 0.16, 0.4, 0.6, 1.0, 1.4, 2.0, 4.0 and 10.0 g) were applied to the hind paws. The 50% withdrawal threshold was calculated using the up-down method as described previously (Chaplan et al., 1994). In some experiments, muscimol (2 μg/μl; 0.5 μl) or ACSF was unilaterally injected into the ACC (AP: +1.0 mm, ML: 0.5 mm, DV: 0.9 mm) under brief isoflurane anesthesia. Mechanical thresholds in the contralateral paws were measured before and 30 min after muscimol injection.

### Statistics

Prism software (GraphPad 7.0, La Jolla, CA, USA) was used to conduct the statistical analysis. Data were presented as mean ± SEM. Tests for differences between two populations were performed using Mann-Whitney test or Wilcoxon signed-rank test as specified in the text. Multiple group comparison was performed with one-way ANOVA followed by a Tukey’s *post hoc* test. Significant levels were set at *P* < 0.05.

## Results

### *In Vivo* Ca^2+^ Imaging of ACC Pyramidal Neurons in Awake Mice

**Figure 1:**
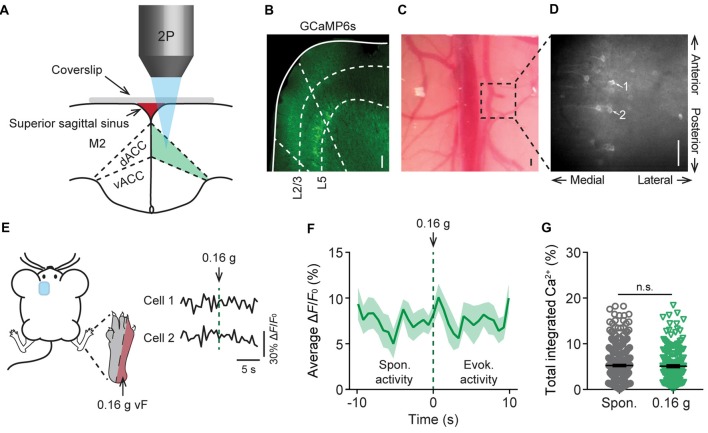
*In vivo* Ca^2+^ imaging of pyramidal neurons in L5 of the anterior cingulate cortex (ACC). **(A)** Schematic diagram showing *in vivo* two-photon imaging in the dorsal ACC (dACC) through a glass window. M2, secondary motor cortex; vACC, ventral ACC. **(B)** Immunostaining of pyramidal neurons expressing GCaMP6s in the cortex of *Thy1*-GCaMP6s transgenic mice. Dashed lines indicate the region of the dACC and the bottom of L2/3 and L5 of the cortex. **(C)** Top-down view of the glass window preparation. Two-photon images were collected from the boxed region. **(D)** *In vivo* Ca^2+^ imaging in the ACC. Noted that apical dendrites of ACC pyramidal neurons point towards the midline of the brain. Scale bars in **(B**–**D)**, 100 μm. **(E)** Representative fluorescence traces from L5 pyramidal cells expressing GCaMP6s in the ACC. Mechanical stimulation was applied using a 0.16 g von Frey (vF) filament to the lateral plantar aspect of the contralateral hind paw. **(F)** Population average response of L5 pyramidal neurons before and after 0.16 g vF stimulation. Stimulation was applied at time 0. Envelop indicates SEM. **(G)** Average total integrated Ca^2+^ activity over 10 s in L5 pyramidal neurons (Spontaneous: 5.2 ± 0.4%, *n* = 305 cells from five mice; 0.16 g-evoked: 5.1 ± 0.4%, *n* = 182 cells from four mice; *P* = 0.87, Mann-Whitney test). Summary data are presented as mean ± SEM.

To study acute nociceptive responses in the ACC, we used *in vivo* two-photon Ca^2+^ imaging to examine the somatic activities of L5 pyramidal neurons expressing a genetically-encoded Ca^2+^ indicator GCaMP6s in awake, head-restrained mice. The neuronal somata in L5 of the ACC are located below the secondary motor cortex, more than 800 μm below the pial surface, and situated within the longitudinal fissure which was largely covered by the superior sagittal sinus and a thick skull. To image these neurons, we performed a craniotomy and implanted a glass window in the center of the skull (see “Materials and Methods” section; Figures 1A,B). Through the glass window, we were able to image GCaMP6s-labeled pyramidal cells in the cortex of *Thy1*-GCaMP6s transgenic mice within ~900 μm from the pial surface. GCaMP6s-expressing neurons in L5 of the ACC were visible at the depth of 800–900 μm below the pial surface, with a distance of 300–500 μm to the midline and an appearance of horizontally projecting dendritic trunks (Figures 1C,D). When animals were under a quiet resting condition, spontaneous Ca^2+^ transients can be detected in the somata of L5 pyramidal neurons (Figures 1E,F). The average total integrated Ca^2+^ activity over 10 s was 5.2 ± 0.4% (305 cells from five mice) at the baseline condition (Figure 1G). When we applied punctate pressure stimuli to the animals’ contralateral hind paw using a 0.16 g vF filament, the total integrated Ca^2+^ activity averaged over 10 s after stimulation was 5.1 ± 0.4% (182 cells from four mice). This was not significantly different from the spontaneous activity (Figures 1E–G). Because 0.16 g vF filament delivered a force that is below the animals’ mechanical paw withdrawal threshold (Supplementary Figure S1), these data suggest that non-noxious stimuli do not induce significant responses in L5 pyramidal neurons in the ACC.

### Acute Pain-Elicited Pyramidal Neuronal Activity

**Figure 2:**
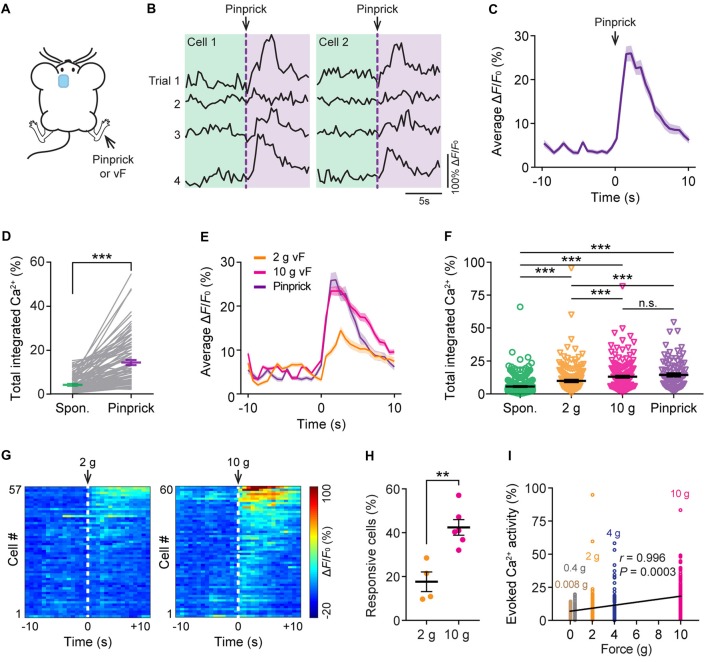
Evoked Ca^2+^ activity of ACC neurons in response to peripheral noxious stimuli. **(A)** Schematic showing *in vivo* Ca^2+^ imaging in the ACC in response to noxious stimulation (pinprick or vF) applied to the contralateral hind paw. vF, von Frey. **(B)** Representative fluorescence traces of L5 pyramidal somata expressing GCaMP6s. Arrow heads indicate the time of pinprick (a total of four trials). **(C)** Population average response of L5 pyramidal somata before and after pinprick (applied at time zero). Envelop indicates SEM. **(D)** Total integrated Ca^2+^ activity of L5 pyramidal somata averaged over 10 s before and after pinprick (93 cells from three mice, ***P < 0.001, Wilcoxon singed-rank test). **(E)** Population average response of L5 pyramidal somata before and after various mechanical stimulation. Envelops indicate SEM. **(F)** Summary quantification of total integrated Ca^2+^ activity of L5 pyramidal neurons averaged over 10 s under various conditions (spontaneous: 5.6 ± 0.3%, 490 cells from 10 mice; 2 g: 9.8 ± 0.8%, 177 cells from four mice; 10 g: 13.1 ± 0.7%, 220 cells from six mice; pinprick: 14.4 ± 1.2%, 93 cells from three mice. ***P < 0.001, one-way ANOVA followed by Tukey’s test). **(G)** Average Δ*F*/*F*_0_ over four trials for individual pyramidal neurons recorded. **(H)** Fractions of ACC neurons responsive to 2 g or 10 g vF stimulation (2 g: 17.7 ± 4.9%, *n* = 4 mice; 10 g: 42.4 ± 3.6%, *n* = 6 mice, **P < 0.01, Mann-Whitney test). Summary data are presented as mean ± SEM. **(I)** Evoked Ca^2+^ activity in ACC neurons correlates with the force of mechanical stimulation (Pearson Correlation, *r* = 0.996, *P* = 0.0003).

To study the neuronal responses in the ACC due to acute pain, we imaged the Ca^2+^ activity of the same neurons before and after noxious stimulation to the animal’s contralateral hind paw. Noxious stimulation was delivered either by a pinprick or via punctate pressure stimuli (2 g or 10 g vF filament; Figure 2A), which induced paw withdrawal behavior (Supplementary Figure S1). We found that pinprick evoked large Ca^2+^ transients in L5 pyramidal neurons in the ACC (Figures 2B,C). The integrated Ca^2+^ activity averaged over 10 s after pinprick was about threefold higher than that at the baseline condition (Figure 2D; *P* < 0.001). Besides pinprick, noxious stimulation with a 2 g or 10 g vF filament also evoked large Ca^2+^ transients in L5 pyramidal neurons in the ACC (Figure 2E). The total integrated Ca^2+^ activity averaged over 10 s following 2 g or 10 g vF stimulation was significantly higher than the spontaneous activity (Spontaneous: 5.6 ± 0.3%, 490 cells from 10 mice; 2 g: 9.8 ± 0.8%, 177 cells from four mice; 10 g: 13.1 ± 0.7%, 220 cells from six mice; pinprick: 14.4 ± 1.2%, 93 cells from three mice; Figure 2F). Moreover, more L5 pyramidal cells showed increased responses to the 10 g vF filament as compared with the 2 g one (Figures 2G,H; *P* = 0.0095). The degree of evoked neuronal activity significantly correlated with the intensity of vF stimulation (Pearson *r* = 0.996; *P* = 0.0003; Figure 2I). Together, these results confirm that ACC pyramidal neurons receive peripheral nociceptive inputs and indicate that neuronal activity in L5 of the ACC correlates with noxious intensity.

### ACC Neurons Show Bilateral Responses to Peripheral Noxious Stimuli

**Figure 3:**
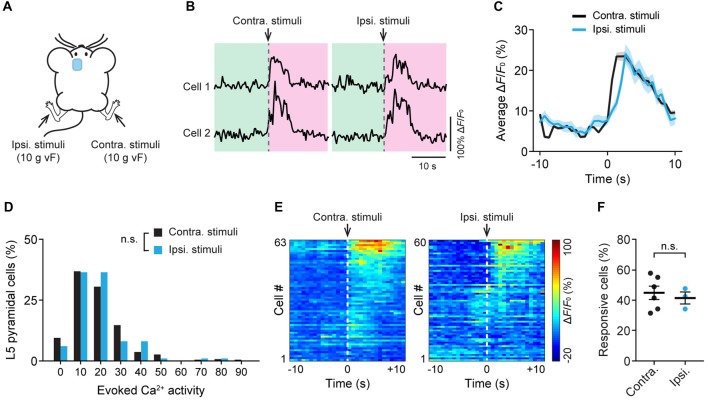
Bilateral responses of ACC neurons to mechanical stimulation to the hind paw. **(A)** Schematic showing *in vivo* Ca^2+^ imaging in the ACC while mechanical stimulation was applied to the contralateral (contra.) or ipsilateral (ipsi.) hind paw. vF, von Frey. **(B)** Fluorescence traces of representative L5 pyramidal somata expressing GCaMP6s. Arrow heads indicate 10 g vF stimulation to the contralateral or ipsilateral paw. **(C)** Population average response of L5 pyramidal neurons before and after contralateral or ipsilateral paw stimulation. Envelops indicate SEM. **(D)** L5 pyramidal soma total integrated Ca^2+^ activity averaged over 10 s after 10 g vF stimulation (contra: *n* = 220 cells from six mice; ipsi: *n* = 99 cells from three mice, *P* = 0.23, Mann-Whitney test). **(E)** Representative heatmap showing Δ*F*/*F*_0_ (average of four trials) of individual ACC neurons from a single animal before and after 10 g vF stimulation to the contralateral or ipsilateral hind paw. **(F)** Fractions of ACC neurons responsive to the contralateral or ipsilateral paw stimulation (contra: 45.3 ± 5.2%, *n* = 6 mice; ipsi: 41.8 ± 3.9%, *n* = 3 mice, *P* = 0.84, Mann-Whitney test). Summary data are presented as mean ± SEM.

Previous studies have shown that acute pain causes bilateral activation of the ACC (Sikes and Vogt, 1992). To further understand the response of L5 pyramidal neurons to noxious stimuli, we recorded neuronal Ca^2+^ activity in the ACC while applying 10 g vF stimulation to the animals’ hind paw on either side (Figure 3A). Similar to what was observed during contralateral paw stimulation, cells in the ACC showed large Ca^2+^ transients when mechanical stimulation was applied to the ipsilateral paw (Figure 3B). The mean peak Δ*F*/*F*_0_ of evoked Ca^2+^ transients was 23.7 ± 1.1% and 24.3 ± 2.4%, respectively, in response to contralateral and ipsilateral paw stimulation (Figure 3C). There was no significant difference in evoked neuronal Ca^2+^ activity between contralateral and ipsilateral paw stimulation (Figure 3D; *P* = 0.23). Furthermore, there was no difference in the percentages of L5 neurons that respond to contralateral or ipsilateral paw stimulation (Figures 3E,F; *P* = 0.84). Together, these results indicate that L5 pyramidal neurons in the mouse ACC exhibit similar activities in response to ipsilateral or contralateral peripheral noxious stimuli.

### Enhanced Spontaneous Activity of ACC Neurons in Mice With Neuropathic Pain

**Figure 4:**
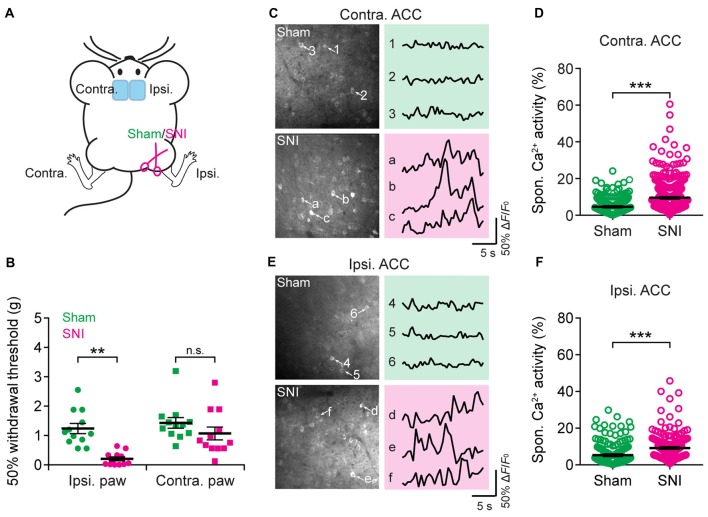
Elevated spontaneous neuronal activity in the ACC of neuropathic pain mice. **(A)** Schematic showing *in vivo* Ca^2+^ imaging in the ACC of sham or spared nerve injury (SNI) mice. **(B)** Mechanical paw withdrawal threshold 2 weeks after sham or SNI surgery (Ipsi: sham: 1.3 ± 0.2 g, *n* = 12 mice; SNI: 0.2 ± 0.07 g, *n* = 12 mice, **P < 0.01, Mann-Whitney test; Contra: sham: 1.4 ± 0.2 g, *n* = 12 mice; SNI: 1.1 ± 0.2 g, *n* = 12 mice, *P* = 0.2). Individual dots represent data from a single animal. **(C)** Representative two-photon images and fluorescence traces of L5 pyramidal neurons expressing GCaMP6s 2 weeks after sham or SNI surgery in the contralateral limb. **(D)** Spontaneous Ca^2+^ activity of L5 pyramidal neurons in the ACC contralateral to the surgical site (sham: 4.6 ± 0.2%, *n* = 270 cells from seven mice; SNI: 9.5 ± 0.3%, *n* = 520 cells from 12 mice, ***P < 0.001, Mann-Whitney test). **(E)** Representative two-photon images and fluorescence traces of L5 pyramidal neurons expressing GCaMP6s 2 weeks after sham or SNI surgery in the ipsilateral limb. **(F)** Spontaneous Ca^2+^ activity of L5 pyramidal neurons in the ACC ipsilateral to the surgical site (sham: 5.3 ± 0.5%; *n* = 191 cells from five mice; SNI: 9.2 ± 0.4%, *n* = 242 cells from six mice. ***P < 0.001, Mann-Whitney test). Individual circles represent data from a single cell. Summary data are presented as mean ± SEM.

Having characterized acute pain-induced neuronal activity in the ACC, we next investigated the activity of ACC neurons in mice with neuropathic pain. Mice were subjected to SNI to induce persistent neuropathic pain (Decosterd and Woolf, 2000; Bourquin et al., 2006; Cichon et al., 2018; Figure 4A). Two weeks after surgery, SNI mice showed mechanical allodynia in the injured hind paw, which was determined by a reduction in the mechanical paw withdrawal threshold. In contrast, sham-operated mice did not show pain hypersensitivity (Figure 4B). We then performed Ca^2+^ imaging in L5 pyramidal neurons in the ACC of SNI and sham mice. We found that the average total integrated Ca^2+^ activity in the somata of L5 neurons under a quiet resting condition was about twofold higher in the ACC contralateral to the SNI site in SNI mice than in sham mice (sham: 4.6 ± 0.2%; 270 cells from seven mice; SNI: 9.5 ± 0.3%, 520 cells from 12 mice; *P* < 0.001; Figures 4C,D). Moreover, the elevation of Ca^2+^ activity in SNI mice was also observed in the ACC ipsilateral to the SNI site (sham: 5.3 ± 0.5%, 191 cells from five mice; SNI: 9.2 ± 0.4%, 242 cells from six mice; *P* < 0.001; Figures 4E,F). The degree of enhanced spontaneous activity after SNI surgery is comparable between both sides of the ACC (*P* = 0.89). These findings indicate that peripheral nerve injury causes an overall increase in the excitability of L5 pyramidal neurons bilaterally in the ACC, even without the noxious input.

### Enhanced Evoked Activity of ACC Neurons in Mice With Neuropathic Pain

**Figure 5:**
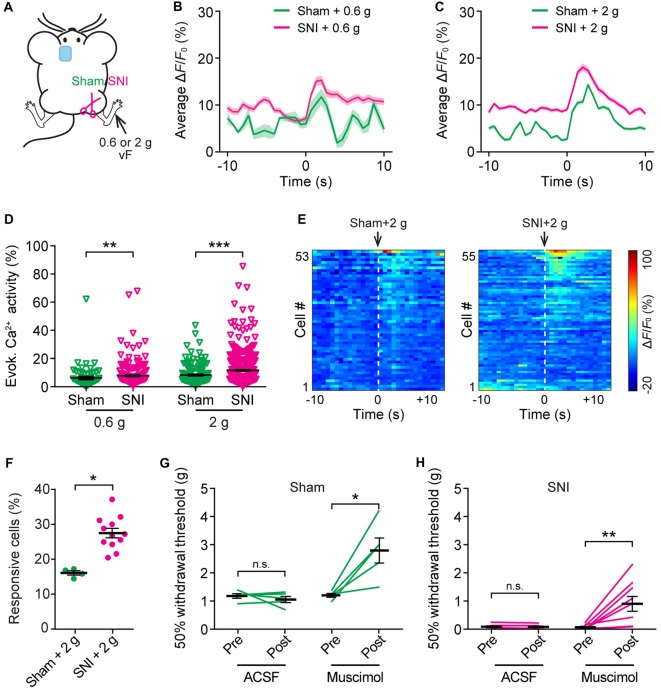
Increased evoked neuronal activity in the ACC of neuropathic pain mice after stimulating the injured limb. **(A)** Schematic showing *in vivo* Ca^2+^ imaging in the ACC of sham or SNI mice 2 weeks after surgery. VF stimulation was applied to the paw of the injured limbs. **(B,C)** Population average response of L5 pyramidal neurons before and after 0.6 g or 2 g vF stimulation. Envelops indicate SEM. **(D)** Mechanical stimulation-evoked pyramidal neuron Ca^2+^ activity in sham or SNI mice (sham + 0.6 g: 6.1 ± 0.1%, *n* = 121 cells from three mice; SNI + 0.6 g: 7.8 ± 0.9%, *n* = 184 cells from four mice; sham + 2 g: 8.7 ± 0.6%, *n* = 161 cells from four mice; SNI + 2 g: 12.4 ± 0.4%, *n* = 520 cells from 12 mice; **P < 0.01, ***P < 0.001, Mann-Whitney test). **(E)** Δ*F*/*F*_0_ (average of four trials) of individual ACC neurons in sham or SNI mice before and after 2 g vF stimulation. **(F)** Fractions of ACC neurons responsive to 2 g vF stimulation in sham or SNI mice (sham: 16.2 ± 0.6%, *n* = 4 mice; SNI: 28.1 ± 1.7%, *n* = 12 mice, *P < 0.05, Mann-Whitney test). **(G)** Paw withdrawal threshold of sham mice before and after artificial cerebral spinal fluid (ACSF) or muscimol (2 μg/μl, 0.5 μl) administration in the ACC (ACSF: Pre: 1.20 ± 0.08 g; Post: 1.07 ± 0.11 g, *n* = 5 mice, *P* = 0.81, Wilcoxon test. Muscimol: Pre: 1.22 ± 0.07 g; post: 2.81 ± 0.44 g, *n* = 5 mice, *P < 0.05, Wilcoxon test). **(H)** Paw withdrawal threshold of SNI mice before and after ACSF or muscimol administration in the ACC (ACSF: Pre: 0.08 ± 0.03 g; Post: 0.08 ± 0.03 g, *n* = 7 mice, *P* = 0.47, Wilcoxon test; Muscimol: Pre: 0.07 ± 0.03 g; Post: 0.9 ± 0.3 g, *n* = 7 mice, **P < 0.01, Wilcoxon test). Summary data are presented as mean ± SEM.

To further understand the nociceptive response of ACC neurons in neuropathic pain, we measured somatic Ca^2+^ activity in L5 pyramidal neurons in response to mechanical stimulation (0.6 g or 2 g vF) applied to the paw surface of the injured limb (Figure 5A). Consistent with the behavioral testing that 0.6 g vF filament caused paw withdrawal responses in SNI mice, but not in sham mice (Supplementary Figure S2), we found that 0.6 g stimulation evoked larger Ca^2+^ transients in SNI mice than in sham mice. The mean peak Δ*F*/*F*_0_ of evoked Ca^2+^ transients in response to 0.6 g vF was 15.3 ± 0.9% in SNI mice and 11.7 ± 1.7% in sham mice (Figure 5B). In response to 2 g vF stimulation, the mean peak Δ*F*/*F*_0_ of evoked Ca^2+^ transients in ACC neurons was 18.1 ± 0.8% and 14.3 ± 0.5%, respectively, in SNI and sham mice (Figure 5C). The total integrated Ca^2+^ activity in L5 pyramidal neurons averaged over 10 s following the mechanical stimulation was significantly higher in SNI mice as compared to that in sham mice (sham + 0.6 g: 6.1 ± 0.1%, 121 cells from three mice; SNI + 0.6 g: 7.8 ± 0.9%, 184 cells from four mice, *P* = 0.008; sham + 2 g: 8.7 ± 0.6%, 161 cells from four mice; SNI + 2 g: 12.4 ± 0.4%, 520 cells from 12 mice, *P* < 0.001; Figure 5D). Furthermore, there were more L5 pyramidal cells responsive to 2 g stimulation in SNI mice than in sham mice (Figures 5E,F; *P* = 0.04). Together, these results reveal enhanced stimulation-evoked activity in ACC neurons in mice with neuropathic pain.

To test the role of ACC hyperactivity in the mechanical allodynia of neuropathic pain mice, we locally administered muscimol (1 μg), an agonist of GABA_A_ receptor, to the ACC of mice contralateral to the surgical site. Mechanical paw withdrawal threshold in SNI or sham mice was measured before and 30 min after muscimol application. We found that administration of muscimol substantially increased the plantar pressure required to elicit paw withdrawal in both SNI and sham mice, whereas injection of ACSF exerted no effects on the animals’ pain behavior (Figures 5G,H).

### Altered Acute Pain Response in the ACC of Neuropathic Pain Mice

**Figure 6:**
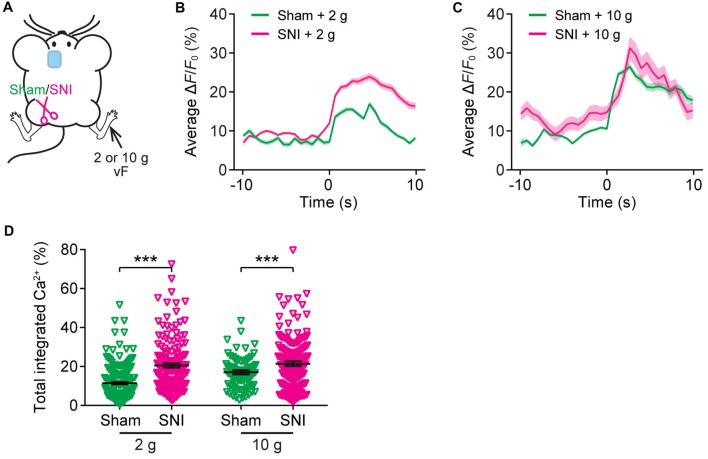
The ACC of neuropathic pain mice shows altered representation of acute pain in the uninjured site. ** (A)** Schematic showing *in vivo* Ca^2+^ imaging in the ACC of sham or SNI mice 2 weeks after surgery. VF stimulation was applied to the paw of the uninjured limbs. **(B,C)** Population average response of L5 pyramidal neurons in sham or SNI mice before and after vF stimulation to the uninjured limb. Envelops indicate SEM. **(D)** Mechanical stimulation-evoked pyramidal neuron Ca^2+^ activity in sham or SNI mice (sham + 2 g: 11.4 ± 0.6%, *n* = 201 cells from five mice; SNI + 2 g: 20.5 ± 1.1%, *n* = 240 cells from five mice; sham + 10 g: 17.0 ± 1.1%, *n* = 99 cells from three mice; SNI + 10 g: 21.4 ± 1.2%, *n* = 102 cells from three mice; ***P < 0.001, Mann-Whitney test). Summary data are presented as mean ± SEM.

Recent *in vivo* multichannel recording studies in rats suggest that neuropathic pain may cause the generalization of the acute pain (Zhang et al., 2017). To test this possibility, we asked whether the elevation of evoked neuronal activity in the ACC of neuropathic pain mice may also occur when mechanical stimulation was applied to the uninjured paw of SNI mice. Here, we imaged Ca^2+^ activity in the ACC of SNI mice when their uninjured paws were stimulated (Figure 6A). We found that SNI mice showed higher evoked Ca^2+^ activity in comparison with sham-operated animals (Figures 6B–D). The mean peak Δ*F*/*F*_0_ of evoked Ca^2+^ transients in response to 2 g vF stimulation was 23.9 ± 0.8% in SNI mice and 16.9 ± 0.7% in sham mice (Figure 6B). The mean peak Δ*F*/*F*_0_ of evoked Ca^2+^ transients in response to 10 g stimulation was 31.3 ± 2.9% in SNI mice and 26.5 ± 0.8% in sham mice (Figure 6C). The total integrated Ca^2+^ activity in ACC neurons averaged over 10 s after stimulation was significantly higher in SNI mice as compared to sham mice (sham + 2 g: 11.4 ± 0.6%, 201 cells from five mice; SNI + 2 g: 20.5 ± 1.1%, 240 cells from five mice, *P* < 0.001; sham + 10 g: 17.0 ± 1.1%, 99 cells from three mice; SNI + 10 g: 21.4 ± 1.2%, 102 cells from three mice, *P* < 0.001; Figure 6D). Together, these results suggest that the response to acute pain stimuli in the ACC pyramidal neurons is altered in mice with chronic pain.

## Discussion

In this study, we performed two-photon Ca^2+^ imaging in awake behaving mice to monitor the activity of individual pyramidal neurons in the ACC, the brain region that mediates affective-aversive responses to painful stimuli (Vogt, 2005; Bliss et al., 2016). We showed that pyramidal neurons located in L5 of the dorsal ACC are responsive to peripheral noxious pain stimuli (e.g., pinprick, >2 g von Frey), but not to non-noxious stimuli (e.g., 0.16 g von Frey). Bilateral activation of the ACC occurred in response to acute pain stimuli applied to either contralateral or ipsilateral paws, and evoked neuronal activity correlated with the noxious intensity. In mice with chronic neuropathic pain, both spontaneous and evoked activities of L5 neurons were elevated in the bilateral ACC. Such pyramidal neuron hyperactivity in the ACC may underlie the animals’ enhanced aversive response to painful stimuli.

Until now, much of the experimental data in pain circuits has come from electrophysiological studies in anesthetized animals or from *ex vivo* or *in vitro* preparations. Given the powerful impact of general anesthetics on synaptic transmission (Franks and Lieb, 1994; Rudolph and Antkowiak, 2004; Huang et al., 2016), the neuronal responses evoked by painful stimuli under anesthetized conditions may not fully represent those occurring in the awake state. To better understand pain-related neuronal responses, we performed* in vivo* Ca^2+^ imaging in the ACC of awake, head-restrained mice using GCaMP6, an indirect reporter of neuronal activity (Chen et al., 2013). GCaMP6s can produce large fluorescence transients (~20% Δ*F*/*F*_0_) in response to single action potentials, and individual spikes within a burst result in stepwise fluorescence increases (Chen et al., 2013). Previous studies using two-photon imaging and patch-clamp electrophysiology have reported a significant correlation between calcium signals and action potential generation in cortical neurons *in vivo* (Smith et al., 2013; Palmer et al., 2014). Although it is difficult to resolve the number of action potentials when neuronal firing rates are high, owing to the long decay time constant of GCaMP6 fluorescence, GCaMP6 offers a great means to observe changes in neuronal activity in large populations of cells and in specific cortical layers. Consistent with previous studies (Cichon et al., 2017), we found a diversity in Ca^2+^ traces in pyramidal cells under a resting condition. Moreover, single noxious pain stimulus evoked large Ca^2+^ transients in L5 pyramidal neurons in the ACC, whereas non-noxious stimuli had no evident effect in the same population of neurons. Importantly, we found that the degree of stimulation-evoked activity in L5 ACC pyramidal neurons correlated with the intensity of mechanical stimulation. These findings, together with previous human imaging studies (Coghill et al., 1999; Büchel et al., 2002), implicate a role of ACC in pain intensity encoding.

Previous electrophysiological recordings in rats (Yamamura et al., 1996; Wang et al., 2003) and rabbits (Sikes and Vogt, 1992) have shown that nociceptive neurons in the ACC have bilateral receptive fields. Consistent with those studies, our Ca^2+^ imaging data show that L5 pyramidal neurons in the ACC were activated by noxious stimulation to either contralateral or ipsilateral paws. There was no obvious contralateral predominance in the ACC during pain processing. This is probably in part due to the bilateral interactions of neurons in the ACC. Indeed, anatomically, L5 ACC pyramidal neurons receive inputs not only from ipsilateral thalamus, but also from ACC neurons in both hemispheres (Allen Brain Atlas^1^).

One important finding in the current study is that peripheral neuropathic pain causes pyramidal neuron hyperactivity in L5 of the ACC. While considerable efforts have been made toward unraveling the plastic changes in the peripheral afferents after nerve damage, much less is known about how cortical neurons are altered in chronic pain conditions. Using *in vivo* Ca^2+^ imaging in awake mice, a recent study reported that peripheral nerve injury caused a persistent elevation in pyramidal neurons’ spontaneous activity in the primary somatosensory cortex (S1; Cichon et al., 2017), a cortical area responsible for the sensory components of pain (Bushnell et al., 1999). With the same approach, the present study showed that in the ACC, L5 pyramidal neurons exhibited a substantial increase in both spontaneous and evoked somatic Ca^2+^ activity after peripheral nerve injury. Because both the ACC and S1 are positioned on the pain ascending pathway and receive nociceptive inputs from medial thalamus (Vogt and Sikes, 2000), the hyperactive states of pyramidal cells in both regions may represent a prominent feature of cortical circuitry after the transition from acute to chronic pain. Indeed, the strategies to dampen either S1 or ACC pyramidal neurons’ hyperactivity or plasticity, have demonstrated benefits against the development of chronic, debilitating pain (Li et al., 2010; Kang et al., 2015; Santello and Nevian, 2015; Kim et al., 2016; Cichon et al., 2017; Santello et al., 2017; Zhang et al., 2017).

Besides the symptoms of allodynia at the site of tissue or nerve injury, recent evidence suggests that chronic pain may cause a generalized enhancement in pain aversion. A recent report in rats showed that chronic inflammatory pain at one site of the body can increase the animals’ aversive response to acute pain stimuli in a separate location (Zhang et al., 2017). Consistent with this study, we found that mice with neuropathic pain exhibited enhanced neuronal activity in the ACC, not only to the painful stimuli applied to the injured limb, but also to pain stimulation applied to non-injured side. This is likely because the ACC receives nociceptive inputs from both contralateral and ipsilateral sides of the body (Sikes and Vogt, 1992), and thus peripheral nerve injury on one side of the body may sensitize neurons located in both sides of ACC, which counts for the exaggerated neuronal response in chronic pain mice regardless of the location of peripheral acute pain signals. These results resonate with the clinical observations that pain experienced by patients with certain pain syndromes, such as chronic postoperative pain and fibromyalgia, can be diffuse (Scudds et al., 1987; Petzke et al., 2003; Kehlet et al., 2006; Kudel et al., 2007; Scott et al., 2010).

In summary, our study provided an *in vivo* characterization of pain-related L5 pyramidal neuronal activity in the ACC of awake mice. Our results indicate that L5 pyramidal neurons in the ACC bilaterally responded to peripheral noxious stimuli, and the development of neuropathic pain was accompanied by an elevation of spontaneous and evoked neuronal activity in both sides of the ACC. The future use of *in vivo* two-photon imaging in awake animals to monitor the activity of specific types of cortical neurons in real time during pain processing may facilitate drug development and target validation for pain treatment.

## Author Contributions

RZ, ZX, JW, W-BG and GY designed research studies. RZ performed *in vivo* imaging experiments. HZ and LH performed sciatic nerve surgery. RZ and HZ performed muscimol injection and mechanical allodynia test. All authors contributed to data interpretation. RZ and GY wrote the manuscript.

## Conflict of Interest Statement

The authors declare that the research was conducted in the absence of any commercial or financial relationships that could be construed as a potential conflict of interest. The reviewer TJP and handling Editor declared their shared affiliation.

## Footnotes

^1^http://connectivity.brain-map.org/
