# Supplementary material for: Neuropathic Pain Causes Pyramidal Neuronal Hyperactivity in the Anterior Cingulate Cortex
Source: Front Cell Neurosci. 2018 Apr 20;12:107. doi: 10.3389/fncel.2018.00107 (PMC5919951; doi:10.3389/fncel.2018.00107)
Supplement: Supplementary file 1 [file Presentation_1.PDF]

Supplementary Material

Neuropathic pain causes pyramidal neuronal hyperactivity in the anterior cingulate cortex

Ruohe Zhao, Hang Zhou, Lianyan Huang, Zhongcong Xie, Jing Wang, Wen-Biao Gan, Guang Yang*

*** Correspondence:** Guang Yang: Guang.Yang@med.nyu.edu

Supplementary Figures

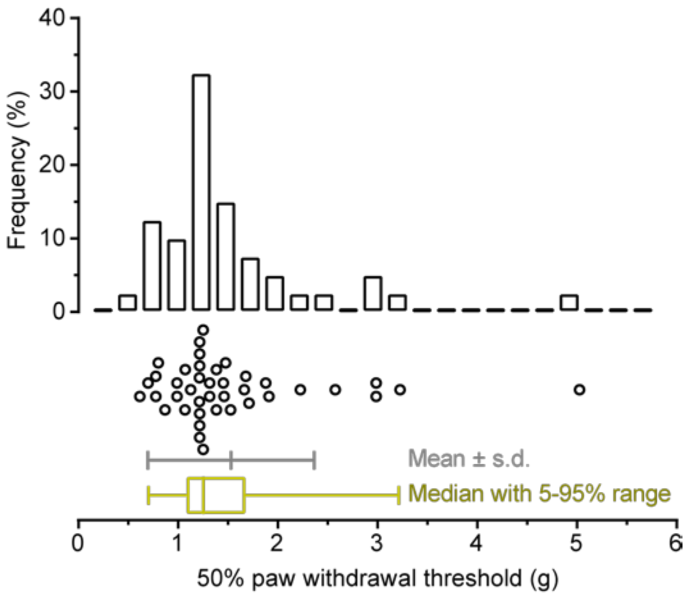

Supplementary Figure 1. Distribution of 50% hind paw withdrawal threshold in normal mice.

Individual circles represent data from a single mouse. The range of mean ± s.d. and 5–95% confidential interval were shown in the bottom panel. Noted that mechanical stimulation with 0.16 g von Frey filament was out of either of these two ranges.

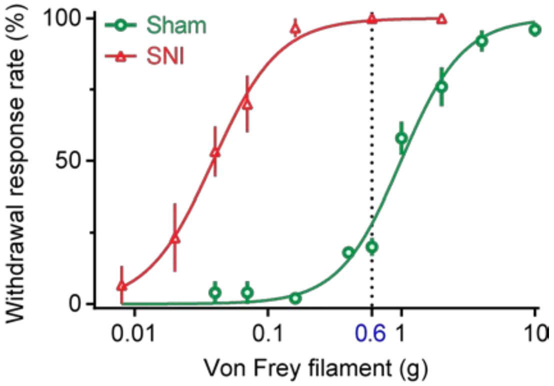

Equation:

$$Y = \frac{100}{1 + \left[\frac{EC50}{X}\right]^h}$$

Parameters of fit

	Sham	SNI
EC50	0.99 g	0.038 g
Hill slope [h]	1.87	1.76
Goodness of fit R^2	0.94	0.92

Supplementary Figure 2. Paw withdrawal threshold of SNI/sham mice with the complete battery of stimuli. Von Frey filaments were presented in ascending order of strength. Each filament was presented 10 times, and the percentage of positive responses was presented as the withdrawal response rate. Equation on the bottom panel was used for curve fitting.
